# Relationship of Serum Levels of IL-17, IL-18, TNF-*α*, and Lung Function Parameters in Patients with COPD, Asthma-COPD Overlap, and Bronchial Asthma

**DOI:** 10.1155/2020/4652898

**Published:** 2020-07-12

**Authors:** Nailya Kubysheva, Marina Boldina, Tatyana Eliseeva, Svetlana Soodaeva, Igor Klimanov, Anastasia Khaletskaya, Venera Bayrasheva, Valery Solovyev, Luis Alfonso Villa-Vargas, Marco Antonio Ramírez-Salinas, Moisés Salinas-Rosales, Dmitry Yu. Ovsyannikov, Ildar Batyrshin

**Affiliations:** ^1^Kazan Federal University, Kremlyovskaya St., 18, Kazan 420000, Russia; ^2^Federal State Budgetary Educational Institution of Higher Education “Privolzhsky Research Medical University, Minin and Pozharsky Square 10/1, Nizhny Novgorod 603005, Russia; ^3^Pulmonology Scientific Research Institute under FMBA of Russia, Orekhovyy Bul'var 28, Moscow 115682, Russia; ^4^State Health Facility “City Clinical Hospital No. 5”, St. Nesterova, 34, Nizhny Novgorod 603005, Russia; ^5^Instituto Politecnico Nacional, Av. Juan de Dios Bátiz, Esq. Miguel Othón de Mendizábal, Col. Nueva Industrial Vallejo, Alcaldía Gustavo A. Madero, 07738 Mexico City, Mexico; ^6^Centro de Investigación en Computación, Instituto Politécnico Nacional (CIC-IPN), Av. Juan de Dios Bátiz, Esq. Miguel Othón de Mendizábal S/N, Gustavo A. Madero, 07738 Mexico City, Mexico; ^7^Department of Pediatrics, Medical Institute, Peoples' Friendship University of Russia (RUDN University), 6 Miklukho-Maklaya St., Moscow 117198, Russia

## Abstract

Determination of markers of systemic inflammation is one of the important directions in the study of pathogenesis and improvement of diagnosis of chronic obstructive pulmonary disease (COPD), asthma-COPD overlap (ACO), and bronchial asthma (BA). The aim of our work was a comparative study of the features of changes in serum levels of IL-17, IL-18, and TNF-*α* in patients with COPD, ACO, and BA with various severity of the disease, as well as evaluation of the relationship between the level of these cytokines and lung ventilation function. A total of 147 patients with COPD (*n* = 58), ACO (*n* = 57), and BA (*n* = 32) during a stable period have been examined in this study. The control group included 21 healthy nonsmokers with similar sex-age indicators. Serum levels of IL-17, IL-18, and TNF-*α* were determined by ELISA. The concentrations of these cytokines in the circulation in the studied patients with COPD, ACO, and BA were higher than those in healthy nonsmokers (*p* ≤ 0.001). IL-17 and IL-18 levels in the blood serum were comparable in all examined patients. The mean TNF-*α* concentrations in the circulation in COPD and ACO were significantly higher than those in BA (*p* < 0.001). In patients with COPD, the levels of IL-17 and TNF-*α* increased progressively against the background of a decrease in numerous spirometric indicators, which allows us to consider these cytokines as systemic biomarkers of disease severity. In BA, the inverse correlations between the level of IL-17 and FEV1/FVC (%) and FEV1 have been found. In patients with ACO, the increase in IL-18 levels was associated with a decrease in FEV1 and TNF-*α* with FEV1/FVC (%). These findings indicate that IL-17, IL-18, and TNF-*α* can participate in the mechanisms of systemic inflammation and the genesis of disorders of airway obstruction in COPD, AСO, and BA. An increase in the levels of IL-17 and TNF-*α* may be associated with impaired bronchial patency in COPD and BA. The established associations of the IL-18 concentration in the blood serum and FEV1 only in patients with ACO allow using the level of IL-18 as a potential marker of the degree of impaired airway obstruction in this disease.

## 1. Introduction

COPD and bronchial asthma are widespread diseases that pose a serious medical and social problem. A key feature of these diseases is the airway obstruction, which is reversible in BA and progressively partially reversible in COPD. The development of morphophysiological changes in COPD and BA is based on the formation of local and systemic chronic inflammation. The inflammatory process in these diseases is characterized by the involvement of a whole ensemble of cellular elements and the secretion of a large array of phlogogenic mediators, both in the respiratory tract and in circulation [1]. The interaction of this complex leads to the proliferation of smooth muscle myocytes and fibroblasts, airway fibrosis and their obstruction, and, ultimately, the progression of the severity of COPD and BA [1, 2]. Airway inflammation in asthma and COPD is markedly different. Asthma is characterized by activation of mast cells and T-helper type 2 cells and eosinophil infiltration in the airway. In COPD, as a rule, there is an infiltration of neutrophils and macrophages, which is caused by activation of Th1, Th17, and T cells of CD8 in the respiratory tract [1, 3]. Many patients show clinical characteristics of both diseases, so-called asthma-COPD overlap (АСО) [4, 5]. Differentiation between asthma, COPD, and ACO is extremely important for determining the pathogenetic features of inflammatory processes in these diseases and taking appropriate therapeutic measures.

Cytokines play a significant role in the formation of inflammation and pathophysiological mechanisms of airway obstruction in both asthma and COPD. TNF-*α* is one of the classic proinflammatory molecules involved in the development of COPD and BA [1, 6]. At the same time, the role of this cytokine in the pathogenesis of ACO requires further study.

At the present stage, experimental data and results of clinical studies on the possible involvement of such phlogogenic molecules as IL-17 and IL-18 in the genesis of airway obstructive diseases have appeared [7, 8]. Interleukin-17 (previously known as IL-17A) is a proinflammatory cytokine. It is known that IL-17 plays a central role in the activation of neutrophilic and macrophage in the lung in patients with COPD and severe BA [3, 9–12]. This cytokine can participate in the contraction of the airway smooth muscles and bronchial hyperreactivity and in the development of emphysema formation [13–16]. However, there are conflicting data on changes in the concentration of this cytokine in patients with COPD [17–19], which emphasizes the importance of studying the role of IL-17 in the development of inflammation of this disease. IL-18 is one of the 11 members of the IL-1*β* family, and like IL-1*β*, it quickly responds to external factors and triggers major proinflammatory reactions [20]. Overexpression of IL-18 in the airways in patients with COPD has been shown and can lead to emphysema, the development of fibrosis in the bronchi and blood vessels of the lungs, and the formation of pulmonary hypertension [21–24]. Due to its Th-2-inducing functions, IL-18 can also participate in inflammation and hyperreactivity of the respiratory tract in asthma and promote the recruitment of eosinophils to the airways [25, 26].

It is worth noting that, despite the available data on changes in the levels of these cytokines in the respiratory tract in patients with COPD and BA, there is little evidence of their participation in the development of systemic inflammation in these diseases and ACO. Also, the role of these proinflammatory mediators in the progression of disease severity and airway obstruction in patients with COPD, ACO, and BA remains poorly understood.

The aim of the study was to compare changes in serum levels of IL-17, IL-18, and TNF-*α* in patients with COPD, ACO, and BA with varying degrees of disease severity and to determine associations of these cytokine concentrations and lung ventilation function.

## 2. Materials and Methods

### 2.1. Study Population

The study included 147 patients with stable COPD, ACO, and controlled BA. Patients visited the outpatient hospital of 28 Nizhny Novgorod. This study was conducted over two years.

The diagnosis of COPD was made in accordance with the recommendations for the diagnosis, treatment, and prevention of this disease (GOLD, 2017) (after evaluating FEV1 and FEV1/FVC obtained during bronchodilation test) [27]. Patients with COPD were divided into four groups according to severity: GOLDI (*n* = 14), GOLDII (*n* = 15), GOLDIII (*n* = 17), and GOLDIV (*n* = 12).

Patients with ACO were diagnosed in accordance with the 2017 GINA/GOLD document [28]: (1) the presence of clinical characteristics of asthma and COPD; (2) indications after bronchodilator spirometry confirming airflow obstruction (FEV1/FVC < 70%); and (3) a postbronchodilatory increase in FEV1 > 12% and 200 ml from baseline.

Patients with asthma-COPD overlap were divided into four groups depending on the severity of airway obstruction: ACOI (*n* = 12)—FEV1 ≥ 80%, ACOII (*n* = 18)—50% ≤ FEV1 < 80%, ACOIII (*n* = 15)—30% ≤ FEV1 < 50%, ACOIV (*n* = 12)—FEV1 < 30%.

Asthma patients were diagnosed according to the recommendations of GINA [29]. Patients with BA had an established diagnosis for more than 5 years. The severity and control level of asthma was determined based on the frequency of day and night symptoms of asthma, the need for short-acting *β*2-agonists, exercise tolerance, FEV1, the variability of FEV1 and peak expiratory flow, and assessment of maintenance therapy. None of them had a history of COPD or previous doctor-diagnosed ACO.

Patients with BA were divided into two subgroups: BA2 (moderate BA, *n* = 20) and BA3 (severe BA, *n* = 12).

Examined patients used medium and high doses of IGS as monotherapy or in combination with LABA in accordance with the severity of the disease.

Exclusion criteria are as follows: ages younger than 40 years, COPD and BA in the acute stage, concomitant diseases such as unstable coronary heart disease, high arterial hypertension, decompensation of heart failure, decompensated diabetes mellitus, exacerbation of gastric ulcer and 12 duodenal ulcer, exacerbation of GERD, systemic connective tissue diseases, malignant tumors, mental illness, and alcohol abuse.

At the time of the study, the individuals examined did not participate in any clinical trials during the last month or at the time of the examination.

The control group included 21 healthy nonsmokers with similar sex and age indicators, who did not take any medications. The healthy subjects had a physical examination in the outpatient hospital and were randomly selected as the control group. Participants in the healthy group did not have any diagnosed respiratory diseases, as evidenced by normal lung function with FEV1/FVC. All healthy subjects in the control group did not have lung disease, diabetes mellitus, coronary heart disease, malignancy, or connective tissue diseases.

The study of spirometric indices (initially and after inhalation of salbutamol 400 *μ*g with a spacer) was carried out using a Spirolab III computer spirometer (Italy).

This work was approved by the Ethics Committee of the Pulmonology Research Institute, Moscow, Russia. The written informed consent was obtained from all participants.

### 2.2. Serum Preparation

The blood samples were obtained in the morning on or before the breakfast from the median cubital vein, then immediately centrifuged at 3,000 rpm for 10 minutes. The serum was extracted and stored at T = -80°C.

### 2.3. Cytokine Concentration Assessment

Cytokine concentrations in the blood serum samples were determined with the use of enzyme immunoassay kits “interleukin-17-EIA-BEST” (the lower limit of detection was 1 pg/ml, measurement range: 0-500 pg/ml), “interleukin-18-EIA-BEST” (the lower limit of detection was 2 pg/ml, measurement range: 0-1000 pg/ml), and “alpha-TNF-EIA-BEST” (the lower limit of detection was 2 pg/ml, measurement range: 0-250 pg/ml) (AO Vector-Best, Novosibirsk, Russia) in accordance with the manufacturer's instructions.

### 2.4. Statistical Analysis

The statistical analysis was carried out using the Statgraphics Centurion software package, v.9. To determine the distribution normality, the Shapiro-Wilk test was used. In the case of a correct distribution, the data are presented as the mean value (*M*) ± standard deviation (*δ*), in the case of an incorrect distribution—in the form of a median (Me) and upper and lower quartiles. When comparing two independent groups of quantitative characteristics in the case of a correct distribution, the Student criterion was used. If the distribution did not correspond to the normal one, the Whitney-Mann *U* test was used when comparing the order signs. For comparing 3 or more groups with the correct distribution, one-way ANOVA was used. To calculate the correlation coefficient (*r*) the Pearson correlation test was used. The differences were considered statistically significant at *p* < 0.05.

## 3. Results

**Figure 1:**
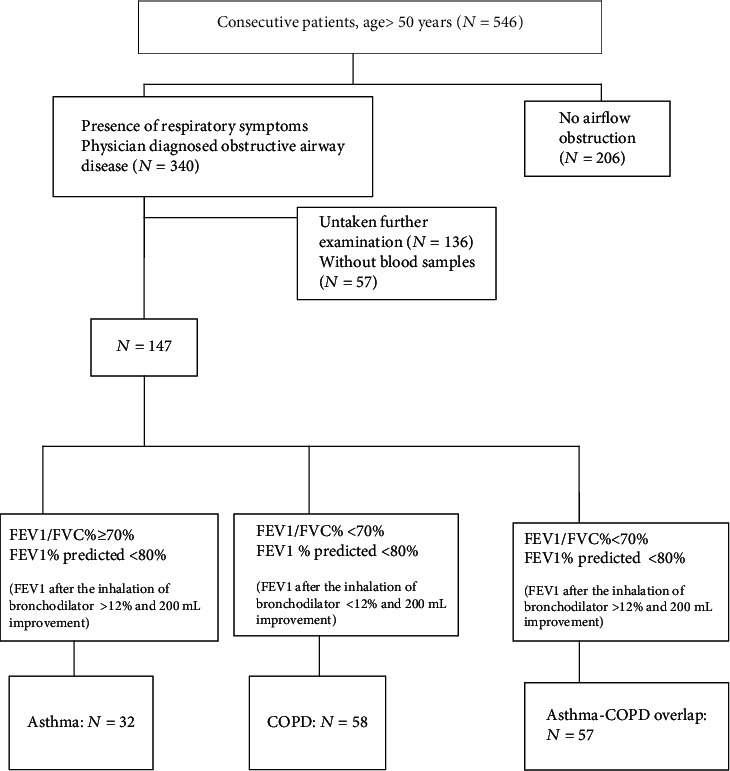
The flowchart of the study population. COPD: chronic obstructive pulmonary disease; FEV1: forced expiratory volume in 1 s; FVC: forced vital capacity.

Figure 1 shows the flowchart of patient recruitment. A total of 546 subjects successfully completed spirometry. 340 subjects had physician-diagnosed airway obstruction, and of these, 147 patients were recommended for further evaluations.

The demographic and functional characteristics of the COPD, ACO, and BA patients are presented in Table 1.

A total of 58 patients with COPD (50 men and 8 women; mean age 63.1 ± 9.6 years), 57 patients diagnosed with ACO (23 men and 34 women; mean age 61.7 ± 8.6 years), and 32 people diagnosed with BA (5 men and 27 women, mean age 58.0 ± 7.9 years) were included in the study.

The difference between the groups according to the sex ratio was statistically significant (*p* < 0.01). The group of patients with COPD was predominantly men (87.8%), and the group of patients suffering from BA was women (84.4%). All patients included in the study were comparable in age (*p* > 0.05).

Active smokers were more likely to be patients with COPD (87.8%). In ACO, smokers were found in 32.6%, and smokers with BA were observed only in 3.1% of cases. 18% of all examined patients with COPD and BA were exposed to professional pollutants.

The FEV1 (% pred) value was higher in BA (75.15 ± 18.76) compared with a group of patients with COPD (55.3 ± 21.2) and ACO (60.8 ± 0.5) (*p* < 0.05). The mean values of FVC (l,% pred) and IC (%) differed in patients with COPD relative to ACO and BA (*p* < 0.05). At the same time, these lung function parameters between patients with ACO and BA were comparable (*p* > 0.05).

**Figure 2:**
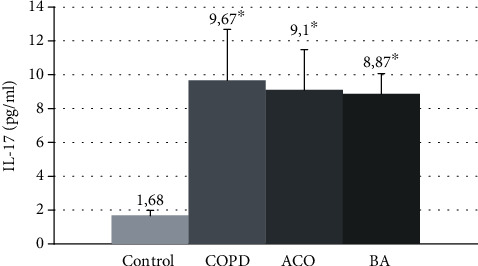
The mean IL-17 concentrations in the blood serum in patients with COPD, asthma-COPD overlap, and bronchial asthma. COPD: chronic obstructive pulmonary disease; ACO: asthma-COPD overlap; BA: bronchial asthma; ^∗^*p* < 0.05 versus healthy nonsmokers.

The mean concentration of IL-17 in examined patient groups was increased by 5.3 times compared with the control (1.68 [1.3; 2.1] pg/ml, ANOVA *p* ≤ 0.0001) (Figure 2). The significant differences in the serum level of IL-17 between the groups of patients with COPD, asthma-COPD overlap, and asthma have not been found (*p* > 0.05).

**Figure 3:**
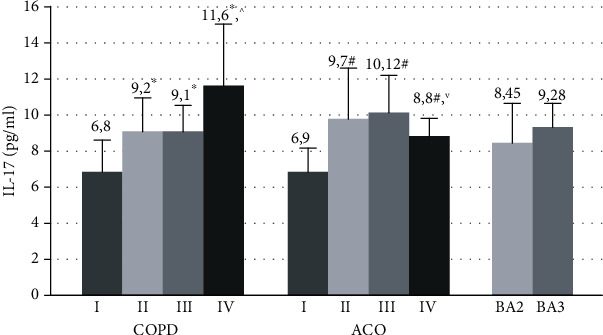
The serum concentrations of IL-17 in patients with COPD, ACO, and BA with varying severity of disease and airway obstruction. COPD: chronic obstructive pulmonary disease; ACO: asthma-COPD overlap; BA: bronchial asthma; I: GOLDI, ACOI; II: GOLDII, ACOII; III: GOLDIII, ACOIII; IV: GOLDIV, ACOIV; BA2: moderate bronchial asthma, BA3: severe bronchial asthma; ^∗^*p* < 0.05 versus patients with GOLDI, ^^^*p* < 0.05 versus patients with GOLDII and GOLDIII; ^∨^*p* < 0.05 versus patients with ACOIV; ^#^*p* < 0.05 versus patients with ACOI.

A comparative study of changes in the concentration of IL-17 in the examined patients with COPD, ACO, and BA showed the following results (Figure 3).

In COPD patients, IL-17 levels in the blood serum tended to enhance as the severity increased. The maximum serum concentration of IL-17 was found in very severe COPD (11.6 [8.6; 18.9] pg/ml), which significantly exceeded values in patients with mild (1.7 times, *p* = 0.001), moderate, and severe diseases (1.3 times, *p* = 0.03).

In GOLDII and GOLDIII patients, the concentrations of this cytokine in the circulation (9.2 [6.6; 12.3] and 9.1 [7.6; 11.5] pg/ml, respectively) did not significantly differ. IL-17 levels in these subgroups were statistically 1.4 times higher relative to GOLDI patients (6.8 [4.5; 9.9] pg/ml, *p* = 0.003).

In ACOI, the serum concentrations of this proinflammatory molecule (6.9 [4.1; 8.7]) was statistically lower than in other patients with this disease (*p* < 0.05). A comparative analysis of changes in IL-17 levels between patients with COPD and ACO revealed that the concentration of this proinflammatory marker in ACOIV was lower (*p* = 0.016) compared to GOLDIV patients.

The IL-17 values in the blood serum in patients with moderate (8.4 [5.3; 11.1 pg/ml] and severe BA (9.28 [6.2; 11.3] pg/ml) were comparable (*p* > 0.05).

**Figure 4:**
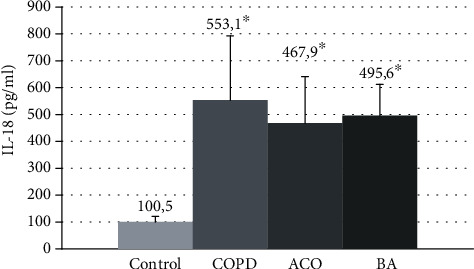
The mean IL-18 concentrations in the blood serum in patients with COPD, asthma-COPD overlap, and bronchial asthma. COPD: chronic obstructive pulmonary disease; ACO: asthma-COPD overlap; BA: bronchial asthma; ^∗^*p* < 0.05 versus healthy nonsmokers.

Evaluation of changes in the mean values of IL-18 in all examined patients showed an excess of the level of this cytokine in patients with COPD, asthma-COPD overlap, and BA compared with healthy individuals by 4-4.8 times (100.5 [68.2; 123, 3 pg/ml, ANOVA *p* ≤ 0.0001) (Figure 4).

The mean concentration of IL-18 between the groups of studied patients with these obstructive diseases did not differ.

**Figure 5:**
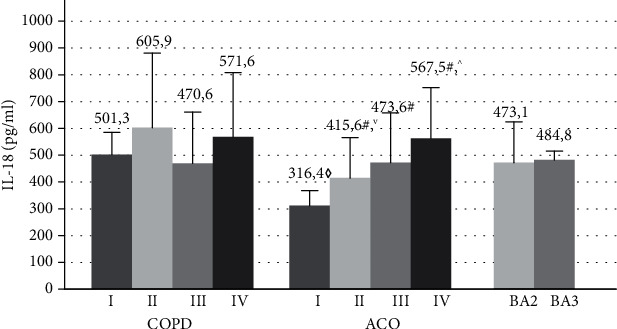
The serum concentrations of IL-18 in patients with COPD, ACO, and BA with varying severity of disease and airway obstruction. COPD: chronic obstructive pulmonary disease; ACO: asthma-COPD overlap; BA: bronchial asthma; I: GOLDI, ACOI; II: GOLDII, ACOII; III: GOLDIII, ACOIII; IV: GOLDIV, ACOIV; BA2: moderate bronchial asthma, BA3: severe bronchial asthma; ^#^*p* < 0.05 versus patients with ACOI; ^^^*p* < 0.05 versus patients with ACOII and ACODIII; ^◊^*p* < 0.05 versus patients with GOLDI; ^∨^*p* < 0.05 versus patients with GOLDII.

The levels of this cytokine in patients with COPD between the subgroups were comparable (*p* > 0.05) (Figure 5).

In the asthma-COPD overlap, the IL-18 level tends to increase against the background of the progression of the severity of the disease (Figure 5). The maximum concentration of this proinflammatory mediator was found in ACOIV (567.5 [309; 839 pg/ml), which was 1.8 times higher than in patients with FEV1 > 80% of this disease (316.4 [272; 372 pg/ml, *p* = 0.01). The serum levels of IL-18 in ACOIII and ACOIII exceeded by 1.3 and 1.5 times the analog values of patients with ACOI (*p* < 0.05).

The significant differences in the level of this cytokine were noted between GOLDI and ACOI (*p* ≤ 0.001), as well as among patients with GOLDII and ACOII (*p* = 0.04). The serum concentrations of IL-18 between subgroups of BA were comparable (*p* > 0.05).

**Figure 6:**
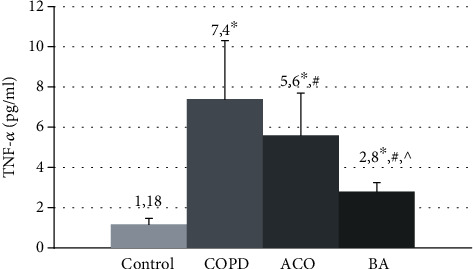
The mean TNF-*α* concentrations in the blood serum in patients with COPD, asthma-COPD overlap, and bronchial asthma. COPD: chronic obstructive pulmonary disease; ACO: asthma-COPD overlap; BA: bronchial asthma; ^∗^*p* < 0.05 versus healthy nonsmokers.

A comparative analysis of the mean values of TNF-*α* showed an increase in the concentration of this cytokine in all examined patients compared with healthy nonsmoking volunteers (1.18 [1.0; 1.9] pg/ml, ANOVA *p* ≤ 0.001) (Figure 6).

The highest mean values of TNF-*α* were observed in patients with COPD (7.4 [2.7; 11.3] pg/ml) and were significantly higher than the control group by 5.1 times (*p* ≤ 0.001). In asthma-COPD overlap, the levels of this proinflammatory cytokine (5.6 [1.62; 10.2 pg/ml) were 3.8 times higher compared to the healthy nonsmokers (*p* ≤ 0.001) and 1.32 times lower than the same values in patients with COPD (*p* = 0.023). In all examined patients with BA, the mean serum concentrations of TNF-*α* (2.8 [2.4; 3.7 pg/ml) were lower relative to patients with COPD and ACO (*p* = 0.001).

**Figure 7:**
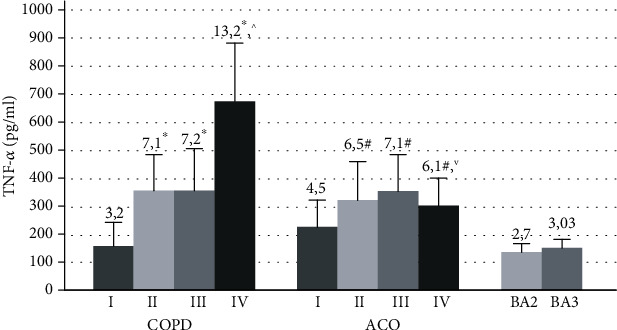
The serum concentration of TNF-*α* in patients with COPD, ACO, and BA with varying severity of disease and airway obstruction. COPD: chronic obstructive pulmonary disease; ACO: asthma-COPD overlap; BA: bronchial asthma; I: GOLDI, ACOI; II: GOLDII, ACOII; III: GOLDIII, ACOIII; IV: GOLDIV, ACOIV; BA2: moderate bronchial asthma, BA3: severe bronchial asthma; ^∗^*p* < 0.05 versus patients with GOLDI, ^^^*p* < 0.05 versus patients with GOLDII and GOLDIII; ^∨^*p* < 0.05 versus patients with ACOIV; ^#^*p* < 0.05 versus patients with ACOI.

TNF-*α* concentration in the circulation in patients with COPD tended to increase with the growing severity of the disease (Figure 7). In GOLDI patients, the serum TNF-*α* levels were lowest (3.2 [2.0; 7.3] pg/ml, *p* < 0.001) compared to other COPD subgroups. The maximum accumulation of systemic TNF-*α* has been found in very severe COPD (13.2 [2.7; 22.6] pg/ml), and its levels significantly exceeded the indicators of other groups (*p* < 0.01).

In asthma-COPD overlap, the serum TNF-*α* concentration in patients with FEV1 > 80% (4.5 [2.5; 10.2] pg/ml) was lower than that in other examined patients with this disease (*p* < 0.05). A comparative analysis of TNF-*α* values in COPD and ACO revealed that the levels of this cytokine in patients with ACOIV was by 2.1 times lower than those in GOLDIV patients (*p* = 0.001).

In bronchial asthma, there were no determinate differences in the serum concentration of TNF-*α* among patients with moderate and severe diseases (Figure 6).

An analysis of the associations between the concentrations of the studied cytokines revealed positive correlations between the levels of IL-17, IL-18, and TNF-*α* in all examined patients with COPD and ACO (Table 2).

We have established a negative correlation between the level of IL-17 and all the investigated parameters of lung function in patients with COPD (Table 3). In this group of patients, inverse associations were registered between the concentration of IL-17 in the blood and FEV1 (%) (*r* = ‐0.55, *p* ≤ 0.001) and FEV1 (l); *r* = ‐0.53, *p* ≤ 0.001).

Also, an increase in the serum levels of IL-17 occurred against the background of a decrease in the high-speed volumetric indicators FEF25, FEF50, and FEF75. A high level of IL-17 in the circulation also correlated with a decrease in values of IC (Inspiratory capacity) (*r* = ‐0.31, *p* = 0.049).

In patients with ACO and BA, significant negative relationships were established between the levels of IL-17 and the values of FVC (%, l) and FEV1 (l). In bronchial asthma, inverse correlations between the serum content of IL-17 and FEV1/FVC (%) were also registered.

There was not found a significant correlation between the level of IL-18 and spirometric indicators in COPD and BA (Table 4). At the same time, in patients with ACO, an increase in IL-18 concentration was associated with a decrease in the values of FVC (%, l) and FEV1 (%).

In patients with COPD, excessive accumulation of TNF-*α* molecules in the circulation was associated with a decrease in many indicators of lung ventilation function: FVC (l,%), FEV1 (l,%), FEV1/FVC (%), FEF25 (%), and FEF50 (%) (Table 5). In ACO, negative correlations between the concentrations of this proinflammatory cytokine and the values of FEV1/FVC (%), FEF50 (%) have been determined. An increase in the TNF-*α* level in the circulation was accompanied by a decrease in FEV1 values (l, %) in patients with BA.

## 4. Discussion

The study of pathogenetic differences in COPD, ACO, and BA is currently relevant. In our work, we studied the features of changes in serum levels of IL-17, IL-18, and TNF-*α* in these diseases. An increase in the serum concentration of these cytokines in COPD, ACO, and BA patients has been found in comparison with healthy nonsmokers. The findings may indicate that IL-17, IL-18, and TNF-*α* can participate in the systemic inflammation of these obstructive airway diseases.

Correlation analysis showed a positive association between the levels of all studied cytokines in patients with COPD and ACO. The revealed associations may signify the identity of the mechanisms regulating the production of these cytokines at the systemic level in patients with COPD and ACO. It is known that phlogogenic cytokines can act synergistically with other proinflammatory molecules and/or induce their synthesis, thereby providing a higher biological effect. For example, it was previously shown that IL-18 could enhance the production of IL-17 [23]. At the same time, one of the important functions of IL-17 is the induction of canonical transcriptional nuclear factor k*β* (NF-k*β*), which can also enhance the inflammatory response and expression of numerous phlogogenic molecules [30].

In our work, a tendency to an increase in the serum concentration of IL-17 with a progression of the severity of the disease has been determined.

The maximum accumulation of this cytokine in the blood serum was determined in patients with very severe COPD. The negative associations between IL-17 levels and all studied spirometric indicators were recorded. These findings imply the participation of IL-17 in the pathogenetic mechanisms of the development of the severity of the course of COPD and form the irreversible processes in the airways in this pathology. It should be noted that COPD is characterized by the irreversible narrowing of the lumen of the airways caused by fibrosis around the small airway [31]. In this case, it is possible that the correlations between the level of IL-17 and such high-speed volumetric indices as FEF25, FEF50, and FEF75 may indicate that this cytokine can also affect the progression of fibrotic processes in the respiratory tract in patients with COPD.

In addition to the presented results, in patients with COPD, the growth of IL-17 levels in the blood serum was accompanied by a decrease in the value of IC (inspiratory capacity). It is known that a decrease in IC reflects the severity of lung hyperinflation or air traps. The development of “air traps” associated with a loss in lung tissue elasticity can lead to decreased expiratory flow in COPD and incomplete lung emptying before the next breath. Thus, the findings may indicate a relationship between the high concentrations of circulating IL-17 and the development of hypoxemia and impaired gas metabolism in COPD.

In contrast to patients with COPD, the significant correlations between IL-17 levels and spirometric parameters in ACO and BA were few. Thus, we believe that IL-17 has the most expressive effect on the degree of violation of airway obstruction in COPD compared with patients with ACO and BA.

The established increase in the level of IL-17 in peripheral blood in these obstructive airway diseases may be associated with the activation of the main cell producer of these cytokines, such as TH17 cells. Several studies have demonstrated an increase in the number of TH17 cells in peripheral blood in patients with COPD [32] and asthma compared to healthy individuals [19]. These cells are mainly associated with neutrophilic inflammation, which is characteristic of patients with COPD. It is known that IL-17 is also able to increase the expression of specific chemokine ligands CXCR1, CXCR2, CXCL8, and GM-CSF, inducing macrophage and neutrophil response in the airway and indirectly activating fibrotic processes in the lung [16, 17]. Thus, probably, IL-17 can indirectly stimulate factors that contribute to the progression of the severity of the disease and form the processes of remodeling in the respiratory tract in these obstructive airway diseases.

The established increase in the IL-18 level in COPD and BA was not associated with a growth in the degree of violation of bronchial patency. In COPD, the high concentrations of this cytokine were typical of both mild and very severe disease. This fact may indicate the participation of IL-18 in the development of systemic inflammation at the early stages of COPD. In ACO, the revealed increase in the level of IL-18 in the blood was associated with a decrease in the FEV1 values (%), which allows taking account of the serum concentration of this cytokine as potential markers of systemic inflammation progression of the disease.

IL-18 has a fairly wide range of biological effects and exhibits pleiotropic properties in the immune response. High expression of this cytokine in the respiratory tract has been shown in patients with COPD and BA [22, 33–36]. At the same time, the role of IL-18 in the implementation of systemic inflammation in obstructive airway diseases is not sufficiently disclosed.

IL-18 serves as a cofactor for the development of both Th1 and Th2 cells and can also increase NK activity and Fas-ligand expression in cells [37, 38]. It is shown that this cytokine exhibits the qualities of a strong coinductor, enhancing the activation of various proinflammatory cytokines, including IFN-g, IFN-*γ*, GM-CSF, TNF-*α*, IL-13, IL-8, IL-17, and IL-5 [23, 39, 40]. It is well known that IL-18, as well as IL-17, stimulating the production of adhesion molecules, can indirectly regulate the accumulation and functioning of macrophages, neutrophils, and eosinophils in the airways [24, 41]. Given the multifaceted proinflammatory nature of IL-18 activity, the detected elevated serum concentrations of this cytokine suggest that IL-18 plays an important role in the implementation of systemic inflammation in both COPD and BA and also in the asthma-COPD overlap.

We revealed a high serum concentration of TNF-*α* in COPD, BA, and AСO compared with healthy people. However, in BA, the mean values of this cytokine were two times more lower than in patients with other obstructive diseases. Perhaps, this proinflammatory mediator makes a more significant contribution to the development of systemic inflammation in COPD and ACO compared with asthma. It is known that TNF-*α* is one of the key proinflammatory cytokines involved in the formation of various pathological processes, including in COPD and BA [1]. The established increase in this mediator in ACO indicates the role of TNF in the development of this disease.

The serum concentration of TNF-*α* in COPD patients tended to increase with growth severity of the disease, which is confirmed by the established numerous correlations between this cytokine level and such spirometric indicators as FEV1 (%), FEV1/FVC, FEF25 (%), and FEF50 (%). In AСO, negative associations were detected between the level of TNF-*α* and only FEV1/FVC, and in BA, between the concentration of this cytokine and FEV1 (%, l). Thus, these associations show that TNF-*α* has the greatest impact on the development of the progression of the severity of the disease in patients with COPD. In addition, it is known that TNF-*α* may not only enhance inflammatory events within the respiratory tract but also plays a role in the development of systemic inflammation; one of the manifestations of which is the development of cachexia in some patients with severe COPD [42].

It should be noted that in ACO patients, changes in the levels of TNF-*α* and IL-17 were similar. It was found that in patients with FEV1 < 30%, the concentration of both TNF-*α* and IL-17 had lower values compared to patients with ACOIII and ACOIII, which may be due to the inhibition of the functional activity of cell producer of these proinflammatory factors in expressive severe inflammation. Moreover, the serum levels of these cytokines in AСOIV were also significantly lower compared to those in patients with very severe COPD. Probably, in this case, the reasons for the distinction found are different pathogenetic mechanisms of the progression of chronic inflammation in these diseases.

Thus, the detected high serum concentrations of IL-17, IL-18, and TNF-*α* in COPD, ACO, and BA may indicate the participation of these cytokines in the development of systemic inflammation in patients with these diseases.

Given that activated immune cells can enter the bloodstream from the airway, the determination of the studied immunomodulatory molecules in the circulation, although indirectly, reflects local pathogenetic processes in these obstructive diseases. The revealed correlations between serum levels of IL-17, IL-18, and TNF-*α* and lung function parameters in the examined patients may signify the effect of these cytokines on the development of pathophysiological mechanisms in these obstructive diseases.

Further studies of systemic inflammation in these patient populations will improve our understanding and provide potential treatment goals for people with obstructive airway diseases.

The strength of this study was that the diagnostic criteria for asthma, COPD, and ACO were based on international recommendations and the clinical characteristics of these diseases.

There are several limitations to the present results. First, many patients have used ICS. It is possible that this treatment could change the levels of the studied cytokines. Secondly, we did not study the changes in the number of eosinophils and neutrophils in the blood and their relationship with the concentration of IL-17, IL-18, and TNF-*α*. Also, in our work, there was no sample size calculation.

## 5. Conclusion

In our work, we found an increase in the serum level of IL-17, IL-18, and TNF-*α* in patients with COPD, ACO, and BA compared with healthy nonsmokers, which may indicate the participation of these cytokines in the pathogenetic mechanisms of development of systemic inflammation of these obstructive diseases.

The established numerous correlations between the parameters of lung function and IL-17 in COPD suggest that IL-17 has the most expressive effect on the severity of airway obstruction in patients with this disease.

The revealed associations of the level of IL-18 in the blood serum and FEV1 only in patients with ACO allow using IL-18 as a potential marker of the degree of impaired bronchial obstruction in this disease.

## Figures and Tables

**Table 1:** Clinical characteristics of the examined patients with COPD, asthma-COPD overlap, and bronchial asthma.

	COPD	ACO	Bronchial asthma	*p*
	1	2	3	
Subjects (*n*/%)	58	57	32	
Men	50 (86,3%)	23 (40%)	5 (15,6%)	*p* = 0.001
Women	8 (13,7%)	34 (60%)	27 (84,4%)	*p*_1−2_ = 0.001 *p*_1−3_ = 0.001 *p*_2−3_ > 0.05
Age (years)	63, 1 ± 9, 6	61, 78 ± 8, 55	58, 0 ± 7, 9	*p* > 0.05
Smokers (abs./%)	51/87,8	18/32,6	1/3,1	*p* = 0.001
Smoking pack-years	36, 0 ± 17, 9	36 ± 17, 9	9	*p*_1−2_ > 05 *p*_1−3_ = 0.001 *p*_2−3_ = 0.001
FEV1 (after bronchodilator) (l)	1, 6 ± 0, 7	1, 5 ± 0, 5	1, 8 ± 0, 54	*p* > 0.05
FEV1 (after bronchodilator) (% pred)	55, 3 ± 21, 2	60, 8 ± 0, 5	75, 15 ± 18, 76	*p*_1−2_ = 0.033 *p*_1−3_ = 0.001 *p*_2−3_ = 0.001
FVC (l)	3, 11 ± 0, 76	2, 77 ± 0, 76	2, 59 ± 0, 78	*p*_1−2_ = 0.02 *p*_1−3_ = 0.003 *p*_2−3_ > 0.05
FVC (% pred)	81, 05 ± 15, 05	88, 59 ± 19, 9	88, 77 ± 13, 7	*p*_1−2_ = 0.02 *p*_1−3_ = 0.001 *p*_2−3_ > 0.05
FEV1/FVC (%)	50, 5 ± 13, 0	53, 31 ± 10, 43	75, 56 ± 5, 1	*p*_1−2_ > 0.05 *p*_1−3_ = 0.001 *p*_2−3_ = 0.001
IC (%)	79, 23 ± 21, 64	90, 98 ± 23, 17	96, 14 ± 30, 76	*p*_1−2_ = 0.01 *p*_1−3_ = 0.001 *p*_2−3_ > 0.05

Data were presented as mean ± SD. COPD: chronic obstructive pulmonary disease; ACO: asthma-COPD overlap; pack-years: number of cigarette packs per day multiplied by the number of smoking years; FEV1: forced expiratory volume in one second; % pred: % predicted; FVC: forced vital capacity, IC: inspiratory capacity (%).

**Table 2:** Correlations between the serum levels of IL-17, IL-18, and TNF-*α* in all examined patients with COPD, asthma-COPD overlap, and bronchial asthma.

	COPD		ACO		BA		All examined patients	
	IL-18	TNF-*α*	IL-18	TNF-*α*	IL-18	TNF-*α*	IL-18	TNF-*α*
IL-17	**r** = 0.49 p ≤ 0.001	**r** = 0.6 *p* ≤ 0.001	**r** = 0.67 *p* ≤ 0.001	**r** = 0.61 *p* ≤ 0.001	*r* = 0.3 *p* = 0.24	*r* = ‐0.23 *p* = 0.6	**r** = 0.54 *p* ≤ 0.001	**r** = 0.56 *p* ≤ 0.001
IL-18		**r** = 0.39 *p* = 0.01		**r** = 0.65 *p* ≤ 0.001		*r* = 0.1 *p* = 0.8		**r** = 0.45 *p* ≤ 0.001

*r*: correlation coefficient; COPD: chronic obstructive pulmonary disease; ACO: asthma-COPD overlap; BA: bronchial asthma.

**Table 3:** Correlations between the serum concentration of IL-17 and the lung function parameters in patients with COPD, asthma-COPD overlap, and bronchial asthma.

Spirometric indicators	COPD		АСО		BA	
	*r*	*p*	*r*	*p*	*r*	*p*
FVC (l)	**-0.51**	**≤0.001**	**-0.42**	**0.022**	**-0.4**	**0.045**
FVC (%)	**-0.35**	**0.02**	**-0.53**	**0.002**	**-0.49**	**0.03**
FEV1 (l)	**-0.53**	**≤0.001**	**-0.48**	**0.007**	**-0.46**	**0.03**
FEV1 (%)	**-0.55**	**≤0.001**	-0.268	0.152	-0.27	0.32
FEV1/FVC (%)	**-0.62**	**≤0.001**	0.031	0.87	**-0.53**	**0.02**
FEF25 (l)	**-0.54**	**≤0.001**	-0.24	0.2	-0.13	0.69
FEF25 (%)	**-0.53**	**≤0.001**	-0.018	0.32	-0.21	0.28
FEF50 (l)	**-0.33**	**0.043**	-0.089	0.64	-0.23	0.21
FEF50 (%)	**-0.53**	**≤0.001**	-0.065	0.73	-0.21	0.27
FEF75 (l)	**-0.56**	**≤0.001**	-0.205	0.27	0.15	0.65
FEF75 (%)	**-0.46**	**0.002**	-0.296	0.11	-0.13	0.7
IC (%)	**-0.31**	**0.049**	-0.211	0.26	-0.2	0.35

*r*: correlation coefficient; COPD: chronic obstructive pulmonary disease; ACO: asthma-COPD overlap; BA: bronchial asthma.

**Table 4:** Correlation between the serum concentration of IL-18 and the lung function parameters in patients with COPD, asthma-COPD overlap, and bronchial asthma.

Spirometric indicators	COPD		ACO		BA	
	*r*	*p*	*r*	*p*	*r*	*p*
FVC (l)	-0.01	0.9	**-0.473**	**0.008**	0.04	0.9
FVC (%)	0.1	0.45	**-0.478**	**0.008**	0.05	0.85
FEV1 (l)	0.107	0.5	-0,19	0,314	0.07	0.81
FEV1 (%)	0.131	0.41	**-0.38**	**0.038**	-0.03	0.9
FEV1/FVC (%)	-0.055	0.72	0.144	0.44	0.2	0.4
FEF25 (l)	0.104	0.51	-.0174	0.35	0.09	0,7
FEF25 (%)	0.118	0.45	-0.107	0.57	0.09	0.7
FEF50 (l)	0.014	0.92	-0.121	0.52	0.25	0.4
FEF50 (%)	0.094	0.55	0.005	0.98	0.29	0.3
FEF75 (l)	0.052	0.74	-0.012	0.95	0.19	0.5
FEF75 (%)	0.058	0.71	-0.204	0.28	0.08	0.78
IC (%)	0.129	0.41	-0.126	0.51	0.2	0.4

*r*: correlation coefficient; COPD: chronic obstructive pulmonary disease; ACO: asthma-COPD overlap; BA: bronchial asthma.

**Table 5:** Correlation between the serum concentration TNF-*α* and the lung function parameters in patients with COPD, asthma-COPD overlap, and bronchial asthma.

Spirometric indicators	COPD		ACO		BA	
	*r*	*p*	*r*	*p*	*r*	*p*
FVC (l)	**-0.32**	**0.03**	-0.36	0.051	-0.25	0.35
FVC (%)	**-0.52**	**0.001**	-0.126	0.51	-0.19	0.53
FEV1 (l)	**-0.318**	**0.04**	0.006	0.97	**-0.47**	**0.03**
FEV1 (%)	**-0.364**	**0.018**	0.192	0.18	**-0.43**	**0.04**
FEV1/FVC (%)	**-0.34**	**0.02**	**-0.405**	**0.02**	-0.21	0.36
FEF25 (l)	-0.279	0.074	0.266	0.15	-0.27	0.33
FEF25 (%)	**-0.313**	**0.044**	0.195	0.18	-0.1	0.73
FEF50 (l)	-0.274	0.079	0.223	0.23	-0.26	0.34
FEF50 (%)	**-0.328**	**0.034**	**-0.361**	**0.05**	0.1	0.74
FEF75 (l)	-0.24	0.12	0.087	0.64	0.25	0.35
FEF75 (%)	-0.267	0.087	0.221	0.24	-0.12	0.7
IC (%)	-0.191	0.226	0.212	0.26	-0.24	0.35

*r*: correlation coefficient; COPD: chronic obstructive pulmonary disease; ACO: asthma-COPD overlap; BA: bronchial asthma.

## Data Availability

The data used to support the findings of this study are available from the corresponding author upon request.
